# Enhanced efficiency of inverted polymer solar cells by using solution-processed TiO_x_/CsO_x_ cathode buffer layer

**DOI:** 10.1186/s11671-015-0754-1

**Published:** 2015-01-31

**Authors:** Xiaodong Zhou, Xi Fan, Xianke Sun, Yunli Zhang, Ziqiang Zhu

**Affiliations:** School of Physics and Electromechnical Engineering, Zhoukou Normal University, Zhoukou, 466001 People’s Republic of China; Hubei Collaborative Innovation Center for Advanced Organic Chemical Materials, Faculty of Physics and Electronic Science, Hubei University, Wuhan, 430062 People’s Republic of China

**Keywords:** Inverted polymer solar cell, Solution processed, Cathode buffer layer

## Abstract

In this work, a double-buffer film of TiO_x_ coated with CsO_x_ (TiO_x_/CsO_x_) was solution prepared to be applied in poly(3-hexylthiophene):indene-C_60_ bisadduct (P3HT:ICBA) and P3HT:[6,6]-phenyl-C_61_-butyric acid methyl ester (PCBM) inverted polymer solar cells (PSCs). Compared with TiO_x_ films and CsO_x_ films, the TiO_x_/CsO_x_ double-buffer film exhibited a favorable energy-level alignment among TiO_x_, CsO_x_, and the electron acceptor of PCBM or ICBA a better surface morphology; and an enhanced wetting and adhesion property with a contact angle of 21.0°, leading to a higher electron mobility of 5.52 × 10^−3^ cm^2^ V^−1^·s^−1^. Moreover, the P3HT:ICBA and P3HT:PCBM photovoltaic devices with the double-buffer film showed the best power conversion efficiency up to 5.65% and 3.76%, respectively. Our results not only present that the double-buffer film is superior than the single film of TiO_x_ and CsO_x_, but also imply that the solution-processed film has a potential to be generally used in roll-to-roll processed organic photovoltaic devices.

## Background

Polymer solar cells (PSCs) have been a hot research topic due to their advantages of low cost, light weight, and large area [1-4]. Recently, normal (not inverted) PSCs with a considerable power conversion efficiency (PCE) of 7% ~ 9% have been reported [5-15]. In such normal PSCs, however, aqueous poly(3,4-ethylenedioxythiophene):poly(styrenesulfonate) (PEDOT:PSS) dispersion is acidic at pH 1 and corrosive to indium tin oxide substrates [16]; In addition, PSS and Al could diffuse into active layers and react with organic active layers [17]; therefore, instability of PSC devices caused by the anode buffer layer of PEDOT:PSS and the Al cathode has become a main concern for practical applications. To overcome the problems, stable inverted PSCs are widely developed by using metal oxides as buffer layers, e.g., zinc oxide (ZnO) [18-20] and titanium oxide (TiO_x_) [21-23] were widely selected as a cathode buffer layer (CBL), whereas MoO_3_ was usually employed as an anode buffer layer to replace PEDOT:PSS and to prevent the diffusing of Al atoms into active layers in inverted PSCs [24].

In inverted PSCs, CBLs play a key role of determining device performance. Generally, for efficient PSCs, a good CBL often satisfies several criteria: high transparency, low work function (WF), and favorable energy levels matched well with those of electron acceptors. 1) High-transparency benefits to large light absorption of active layers, thereby leading to more exciton dissociation at the interface of donor/acceptor and an increase in short current density (*J*_SC_). 2) As reported in previous literatures [25-27], open circle voltage (*V*_OC_) is determined mostly by the energy-level difference between the highest occupied molecular orbital (HOMO) of the donor and the lowest unoccupied molecular orbital (LUMO) of the acceptor [11,12] and the work function difference of the cathode/anode [25-27], as well as the weight ratio of the donor and acceptor [28]. Low work functions of film could increase the work function difference of the cathode/anode and thus leading to an increase in *V*_OC_ of PSCs [25-27]. Afterwards, the films of low WFs facilitate electron collection by cathodes and restrain the charge carrier recombination at the interface of the active layer and film [25,27]. 3) Energy levels of the films, matched well with LUMO and HOMO energies of the components of active layers, could effectively select electrons and block holes, leading to an increase in *J*_SC_ of PSCs [18-23]. However, is there another factor of the films that has a significant effect on the device performance? Will the contact property of interfaces between active layer droplets and cathode buffer layers be changed when the buffer layer is modified? How will the changes of the interfacial contact property affect the charge carrier mobility and the device performance?

**Figure 1:**
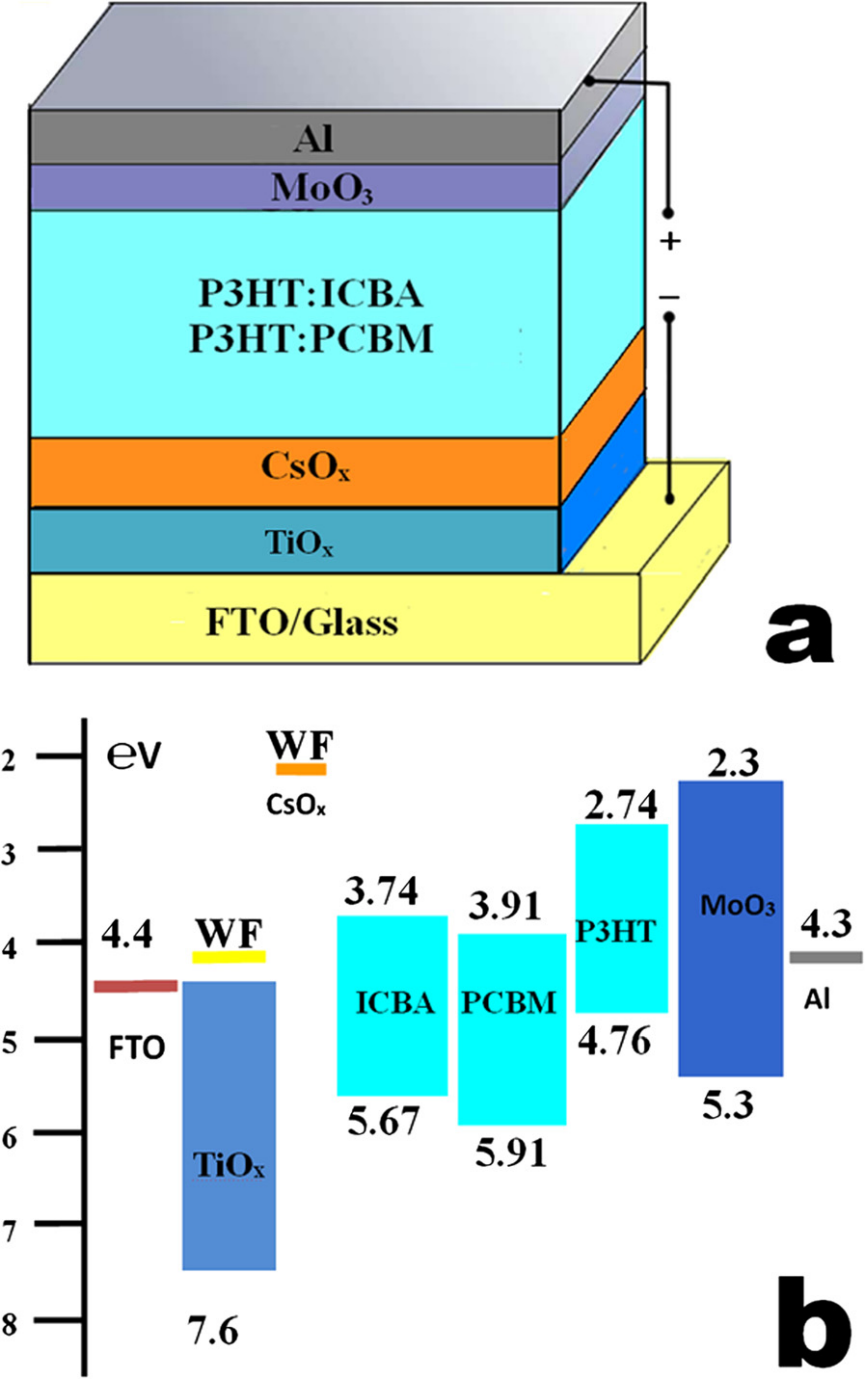
**Device structure and schematic energy diagram. (a)** Device structure of the P3HT:ICBA inverted PSCs and the P3HT:PCBM inverted PSCs. **(b)** Schematic energy diagram of the materials involved in the inverted PSCs.

Recently, many films, such as TiO_x_ [21-23], ZnO [18-20], cesium oxide (CsO_x_) [29], Ca [30,31], LiF [32], and self-assembled monolayers [18,33], are widely employed to modify the cathode surfaces in inverted PSCs. Among these films, much attention in the development of inverted PSCs has been focused on TiO_x_, which has the advantages of excellent chemical and thermal stability, environmentally friendly, high-electron mobility, and easy fabrication [34]. The TiO_x_ film is often prepared by sol-gel synthesis [22], atomic layer deposition [23], and thermal-annealed titanium chelate [16]. Moreover, the film can serve as an effective hole- and exciton-blocking layer because of its conduction band of approximately 4.4 eV, which is much higher than the HOMO values of electron acceptor materials [35,36] (seen in Figure 1b). However, challenges still remain for the film, mainly due to the film work function of 4.14 to 4.22 eV [37] still not being low enough for a high *V*_OC_ in inverted PSCs. The work function commonly affects the work function of cathode and the work function difference of cathode/anode. Thus, the *V*_OC_ of poly(3-hexylthiophene):[6,6]-phenyl-C_61_-butyric acid methyl ester (P3HT:PCBM) inverted PSCs with a TiO_x_ film was usually limited to a small range of 0.55 to 0.58 V under simulated 100-mW cm^−2^ (AM 1.5 G) solar irradiation [23,38], which blocks its practical application in high-efficiency inverted PSCs.

Besides, a CsO_x_ thin film is commonly prepared by evaporating deposition of Cs_2_CO_3_ particles or spin coating the Cs_2_CO_3_ solution. Attributed to the CsO_x_ is a much lower WF of approximately 2.20 eV as reported in previous literature [27]; it not only can modify the work function of the cathode and cathode buffer layer, but also facilitates electron transportation from electron acceptor materials to the CsO_x_ surfaces. Therefore, it is believed that CsO_x_ could be an effective cathode buffer layer for PSCs.

In the work, a solution-processed CsO_x_ film was inserted at the interface of the active layer/TiO_x_. The MoO_3_ film with a thickness of 8 to 10 nm was found to be an optimized value [39]. Here, the Al modified by a 10-nm-thick MoO_3_ film was evaporated. By increasing the work function difference of the cathode/anode, a larger *V*_OC_ and an enhanced PCE were achieved in P3HT:indene-C_60_ bisadduct (ICBA)-based inverted PSCs and in P3HT:PCBM-based inverted PSCs. First, atomic force microscopy (AFM) measurements present that the double film exhibits a smoother surface with a roughness of just 4.9 nm, as compared with the TiO_x_ film and the CsO_x_ film. And, the double film provides a better adhesion with P3HT:ICBA blend solutions, evidenced by measurements of solution contact angles, which was found at the interface between P3HT:ICBA blend droplets and the CBLs. Afterwards, it is found that the highest electron mobility (*μ*_e_) of 5.52 × 10^−3^ cm^2^ V^−1^·s^−1^ is achieved in inverted electron-only devices with TiO_x_/CsO_x_ film measured with space-charge-limited current (SCLC) method. Moreover, current density-voltage (*J-V*) measurements show that the P3HT:ICBA inverted PSCs and the P3HT:PCBM inverted PSCs with TiO_x_/CsO_x_ film exhibit a PCE of 5.65% and 3.76%, respectively, under the illumination of AM 1.5, 100 mW cm^−2^, which is higher than that of the PSCs with TiO_x_ film and the PSCs with CsO_x_ film. The results indicate that the TiO_x_/CsO_x_ is superior than the TiO_x_ and the CsO_x_, not only for the better interfacial contact, but also for the achievement of the higher electron mobility, and thereby leading to an enhanced device performance. Finally, the TiO_x_/CsO_x_ film possesses many advantages, such as 1) solution processability with ethanol and isopropanol solvents, which promote the application of solution-processing technologies, e.g., spin coating and role-to-role printing and 2) low cost since both TiO_x_ and CsO_x_ are cheap to produce and commonly used materials in organic photovoltaic and light-emitting fields, which suggests their huge potential for practical applications.

## Methods

P3HT (4002-E) and PCBM were purchased from Rieke Metals Inc. (Lincoln, NE, USA) and Nano-C (Westwood, MA, USA), respectively. Indene-C_60_ bisadduct was purchased from Solarmer Inc. (El Monte, CA, USA). The TiO_x_ film was prepared by spin coating TiO_x_ sol-gel solution [22] on fluorinated tin oxide (FTO) substrate and then was thermally treated at 200°C for 30 min in air. Whereas, the CsO_x_ film was prepared by spin coating isopropanol solution of Cs_2_CO_3_ on FTO substrate and then thermal annealing at 160°C for 10 min in a glove box filled with Ar atmosphere. When spin coating the Cs_2_CO_3_ solution on FTO/TiO_x_ substrate and then thermal annealing at 160°C for 10 min, it forms the TiO_x_/CsO_x_ film.

All the inverted PSCs were fabricated on FTO-coated glass. First, the different film was spin coated and then baked on FTO. Then, the blend solution of P3HT:PCBM and P3HT:ICBA in dichlorobenzene (1:1, *w*/*w*, 36 mg ml^−1^) was spin coated at 800 rpm. The active layers were then placed into glass petri dishes to undergo solvent annealing and annealed at 150°C for 10 min on a hot plate in a glove box. Subsequently, MoO_3_ (10 nm) and Al (100 nm) were evaporated as an anode buffer layer and anode, respectively, under the pressure of ≤1.0 × 10^−4^ Pa. Transmittance spectra were taken on a Hitachi U-3010 UV-visible spectrophotometer (Hitachi, Ltd., Chiyoda-ku, Japan). The surface morphology of active layers was characterized by AFM (SPM-9500J3, Shimadzu, Kyoto, Japan). The *J*-*V* measurement of the inverted PSCs was conducted on a computer-controlled Keithley 236 Source Measure Unit (Keithley Instruments, Inc., Cleveland, OH, USA). Device characterization was carried out in a glove box under illumination of AM 1.5 G, 100 mW cm^−2^ using a xenon-lamp-based solar simulator (from Newport Co., LTD., Irvine, CA, USA).

## Results and discussion

To investigate the effect of the cathode buffer layers on the performance of the inverted PSCs, we designed six types of inverted PSC devices with different structures:

(A). FTO/CsO_x_/P3HT:ICBA (200 nm)/MoO_3_ (10 nm)/Al (100 nm),
(B). FTO/TiO_x_(80 nm)/P3HT:ICBA (200 nm)/MoO_3_ (10 nm)/Al (100 nm),
(C). FTO/TiO_x_(80 nm)/CsO_x_/P3HT:ICBA (200 nm)/MoO_3_ (10 nm)/Al (100 nm),
(D). FTO/CsO_x_/P3HT:PCBM (200 nm)/MoO_3_ (10 nm)/Al (100 nm),
(E). FTO/TiO_x_ (80 nm)/P3HT:PCBM (200 nm)/MoO_3_ (10 nm)/Al (100 nm),
(F). FTO/TiO_x_(80 nm)/CsO_x_/P3HT:PCBM (200 nm)/MoO_3_ (10 nm)/Al (100 nm).

### Device performance

**Figure 2:**
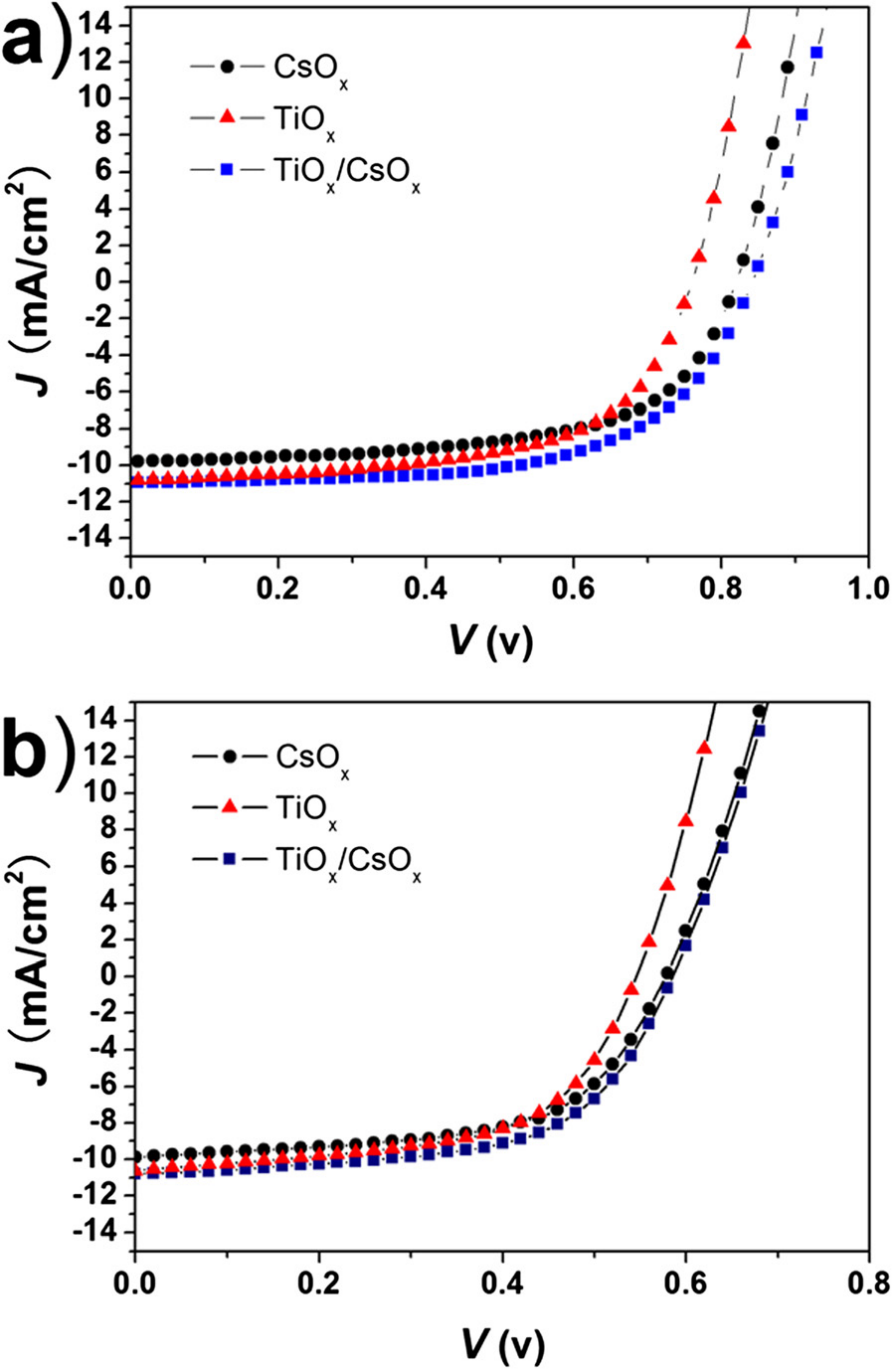
***J-V*** **characteristics of the P3HT:ICBA-based inverted PSCs (a) and the P3HT:PCBM-based inverted PSCs (b) with different film, respectively.**

Figure 2a shows the *J-V* characteristic curves of the P3HT:ICBA inverted PSCs with a film of TiO_x_, CsO_x_, and TiO_x_/CsO_x_ under simulated AM 1.5 G solar illumination of 100 mW cm^−2^. For comparison, more than 30 solar cells were fabricated and characterized to confirm the performance trends. It presents that the inverted PSCs with CsO_x_ film (devices A) show a relatively poor PCE of 4.91% with *V*_OC_ of 0.82 V, *J*_SC_ of 9.79 mA cm^−2^, and fill factor (*FF*) of 61.2%. Compared with the devices A, the PSCs with TiO_x_ film (devices B) yield an equipotent PCE of 4.95%, with a lower *V*_OC_ of 0.76 V, a higher *J*_SC_ of 10.82 mA cm^−2^, and a *FF* of 60.2%. It is considered that the higher *J*_SC_ of 10.82 mA cm^−2^ is attributed to the exciton- and hole-blocking ability of the TiO_x_ film resulted from its favorable conduction band, as shown in Figure 1b. For the PSCs with the TiO_x_/CsO_x_ film (devices C), the highest PCE of 5.65% is achieved with *V*_OC_ of 0.84 V, *J*_SC_ of 10.95 mA cm^−2^, and *FF* of 61.4%, demonstrating a good combination of TiO_x_ and CsO_x_, which compensates the loss in *V*_OC_ of devices B as well as in *J*_SC_ of devices A, respectively. Such photovoltaic performance parameters of the inverted PSCs are summarized in Table 1.

**Table 1:** **Summary of performance of the P3HT:ICBA inverted PSCs and the P3HT:PCBM inverted PSCs with different film**

**Device**	***V***_**OC**_ **(V)**	***J***_**SC**_ **(mA cm** ^**−2**^ **)**	***FF*** **(%)**	**PCE (%)**	**Contact angle (°)**	***μ***_**e**_ **(cm** ^**2**^ **V** ^**−1**^ **·s** ^**−1**^ **)**
CsO_x_^a^	0.82	9.79	61.2	4.91	24.5 ± 0.5	3.85 × 10^−3^
TiO_x_^a^	0.76	10.82	60.2	4.95	32.0 ± 2.0	5.00 × 10^−3^
TiO_x_/CsO_x_^a^	0.84	10.95	61.4	5.65	21.0 ± 0.5	5.52 × 10^−3^
CsO_x_^b^	0.58	9.86	59.6	3.41	---	---
TiO_x_^b^	0.55	10.63	57.3	3.35	---	---
TiO_x_/CsO_x_^b^	0.59	10.81	59.0	3.76	---	---

^a^PSCs based on P3HT:ICBA; ^b^PSCs based on P3HT:PCBM.

To further investigate the general suitability of the TiO_x_/CsO_x_ film in inverted PSCs, the other electron acceptor material of PCBM was used instead of ICBA for fabricating P3HT:PCBM inverted PSCs. The *J-V* characteristic curve is shown in Figure 2b. As expected, for the inverted PSCs with CsO_x_ film (devices D), a PCE of 3.41% is achieved with *V*_OC_ of 0.58 V, *J*_SC_ of 9.86 mA cm^−2^, and *FF* of 59.6%. Compared with that of the devices D, the PCE and *FF* of the inverted PSCs with TiO_x_ film (devices E) just change a little, whereas the *J*_SC_ is enhanced significantly from 9.86 to 10.63 mA cm^−2^ and the *V*_OC_ drops severely from 0.58 to 0.55 V. The inverted PSCs with TiO_x_/CsO_x_ film (devices F) exhibit a PCE of 3.76%, better than that of the devices D and the devices E, which may be due to more electron extraction from the P3HT:PCBM active layer to the FTO cathode. Note that compared with the devices D, the devices E yield an enhanced short-circuit current, maybe due to a better hole-transporting and electron-blocking property of the TiO_x_ than that of the CsO_x_. When TiO_x_/CsO_x_ was used as a cathode buffer layer, it did not induce an increase in *J*_SC_; however, a significant increase in *V*_OC_ from 0.76 to 0.84 V was observed clearly, attributed to the insert of CsO_x_ film with a low work function. The changes in *J*_SC_ and *V*_OC_ of the P3HT:PCBM inverted PSCs agree with those of the P3HT:ICBA inverted PSCs.

### Optical properties and surface morphology of the films

**Figure 3:**
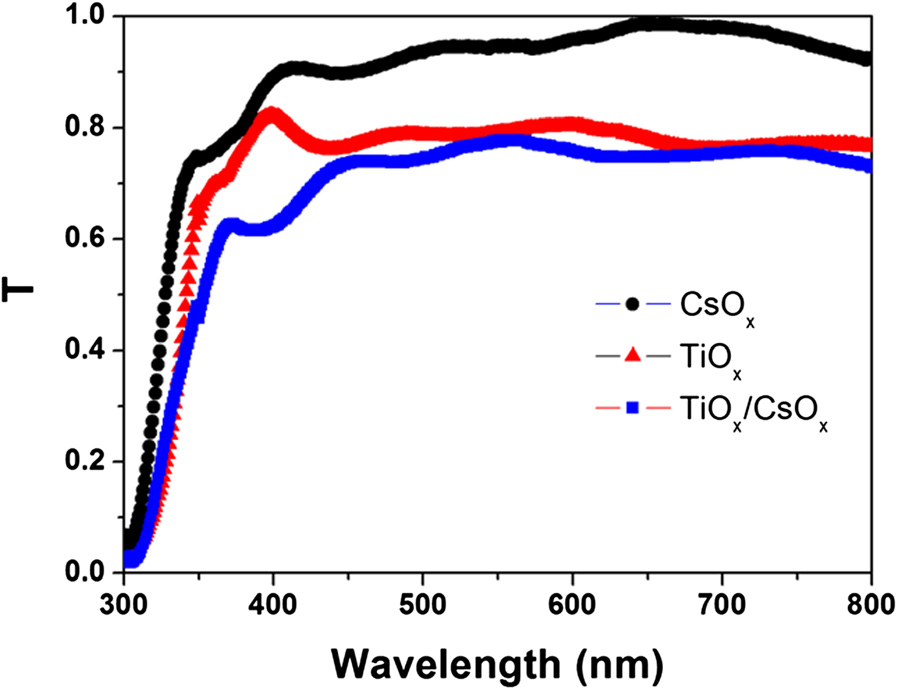
**Optical transmittance of the films of TiO**_x_ **, CsO**_x_ **, and TiO**_x_ **/CsO**_x_ **, respectively.**

Figure 3 shows the optical transmittance of CsO_x_, TiO_x_, and TiO_x_/CsO_x_ on FTO substrates. The CsO_x_ film is highly transparent in the visible range, and the minimum light transmittance is not less than 90% between 400 and 800 nm. Compared with the CsO_x_ film on FTO substrate, the TiO_x_ film exhibits a decreased optical transmittance in the range of 300 to 800 nm, whereas the TiO_x_/CsO_x_ has a decrease optical transmittance of 350 to 450 nm, as compared with the TiO_x_, suggesting an ultra-thin film of CsO_x_ on the TiO_x_ surface.

**Figure 4:**
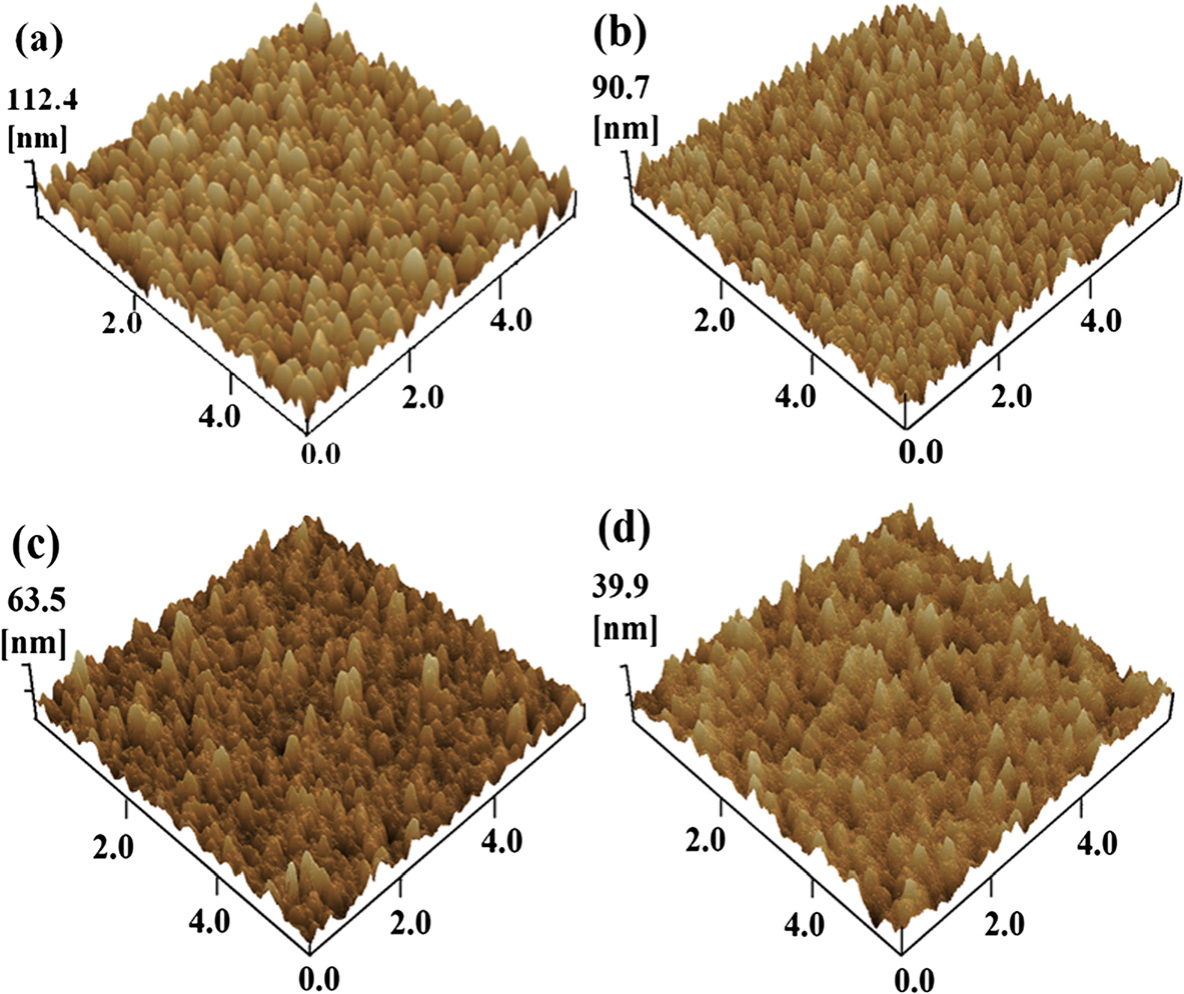
**Surface morphology of FTO substrate (a), TiO**_x_ **(b), CsO**_x_ **(c), and TiO**_x_ **/CsO**_x_ **(d), respectively.** The scan size is 6 μm × 6 μm.

To investigate the surface morphology of FTO modified by the film, atomic force microscopy measurements were carried out. Figure 4 shows the surface images of the four samples, including the FTO substrate, TiO_x_, CsO_x_, and TiO_x_/CsO_x_ film on FTO substrate. It presents that the FTO substrate without any modification by CsO_x_ or TiO_x_ shows a lot of large ‘valleys’ and the root mean square (RMS) is about 15.7 nm. After spin coating Cs_2_O_3_ solution on the FTO substrate and then thermal annealing, it forms a CsO_x_ thin film, which exhibits a lower RMS of 12.5 nm; however, there is not any apparent change in surface morphology between the FTO and the CsO_x_-modified FTO. Due to the modification of TiO_x_ on FTO substrate, the TiO_x_ film exhibits a decreased RMS of about 7.6 nm. Moreover, it shows smaller ‘valleys’ on the surface and becomes much smoother than the CsO_x_ film, whereas the TiO_x_/CsO_x_ film presents a RMS of just 4.9 nm, indicating the CsO_x_ combines well with the TiO_x_ film.

Note that it is difficult to directly measure the thickness of the CsO_x_ film on the top of TiO_x_ film because the thickness of the CsO_x_ film is much smaller than the RMS of either the FTO substrate or the TiO_x_ film. However, the varieties of surface morphology in the four samples have been probed by AFM measurements, implying the presence of the CsO_x_ layer on FTO substrates as well as TiO_x_-modified FTO substrates.

### Droplet contact angle measurements

**Figure 5:**
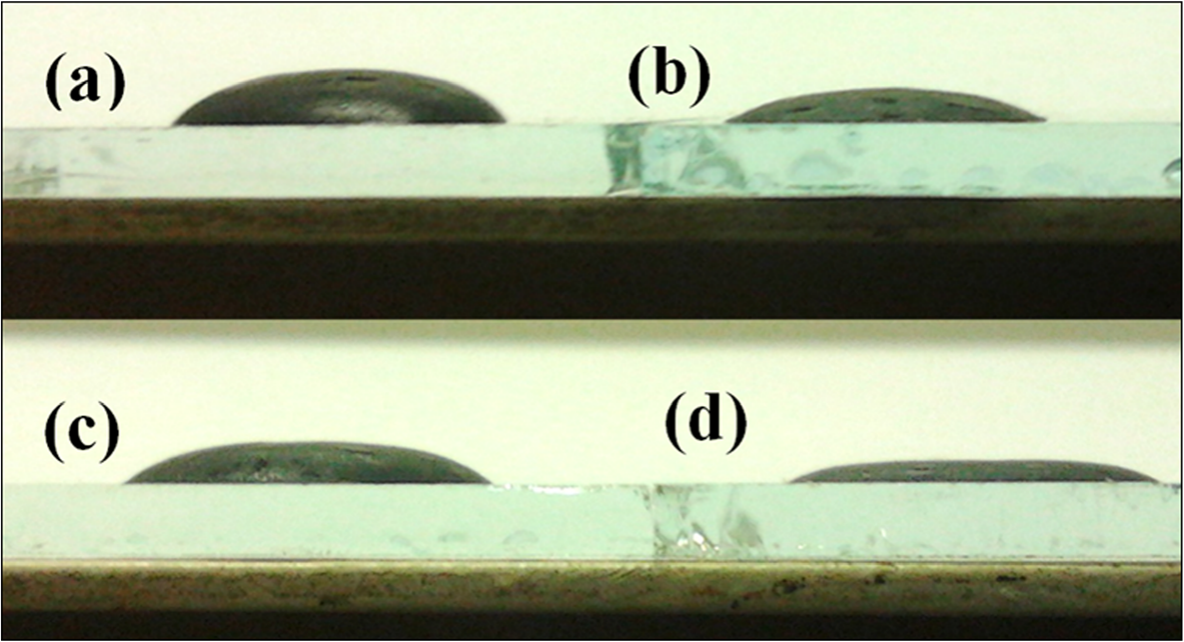
**Droplet images of P3HT:ICBA solution.** Droplet images of P3HT:ICBA solution on the surface of FTO substrate **(a)**, CsO_x_ **(b)**, TiO_x_ **(c)**, and TiO_x_/CsO_x_ **(d)**, respectively. The length of the FTO-coated glass substrate is 3.0 cm.

Due to the device performance being significantly dependent on the interfacial contact property of the film/active layer [40], which contributes to surface energy, it is necessary to observe the surface energy of the film. In this work, the surface energy of the film is studied with measurements of solution contact angles between a drop of P3HT:ICBA solution and the different film. For observing distinctly the interfacial contact angles, we used directly the P3HT and ICBA blend solution (1:1, *w*/*w*, 36 mg ml^−1^) in dichlorobenzene as the ‘solution’ and measured the contact angles between the solution drop and the different film. Figure 5 shows the droplet images of P3HT:ICBA solutions on FTO substrate, CsO_x_, TiO_x_, and TiO_x_/CsO_x_ film, respectively. It presents that the contact angle of the FTO surface to the P3HT:ICBA blend solution is 46.5° ± 2.5°, indicating a poor binding of the blend droplet to the FTO surface. Moreover, compared with the droplet on the FTO substrate, the smaller contact angle of 32.0° ± 2.0° and 24.5° ± 0.5° is observed for the droplet on the TiO_x_ and the CsO_x_ film, respectively, indicating a better wetting of the solvent, whereas the blend droplet has the smallest contact angle of just 21.0° ± 0.5° to the TiO_x_/CsO_x_ film, suggesting the best affinity of the P3HT:ICBA active layer to the TiO_x_/CsO_x_ film, which will be electrically/energetically favorable to the electron extraction and hence facilitates the fabrication of efficient inverted PSCs [29].

The RMS value of the TiO_x_, CsO_x_, and TiO_x_/CsO_x_ is 12.5, 7.6, and 4.9 nm, respectively, and the corresponding P3HT:ICBA solution contact angle is 32.0° ± 2.0°, 24.5° ± 0.5°, and 21.0° ± 0.5°, respectively, suggesting that the contact angle decreases with the decrease of the RMS value. The change of the solution contact angle is partially resulted from the morphology variation induced by the CsO_x_ modification. In the work, the factor of RMS alters the interfacial contact angles and induces an enhancement of wetting and adhesion by changing surface energy, as also reported in previous literature [40].

### Electron mobility measurements

**Figure 6:**
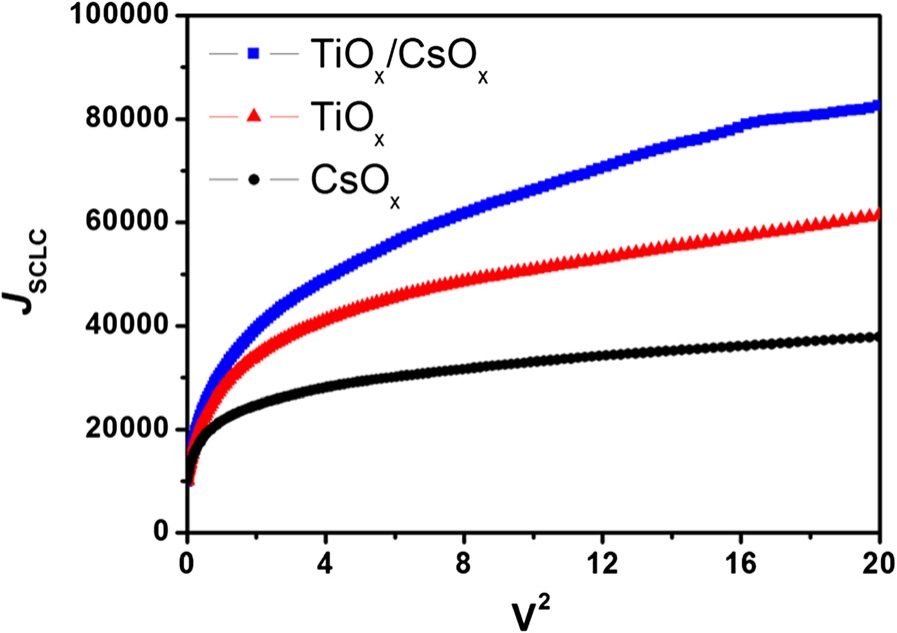
**Corresponding relationship of** ***J***_SCLC_ **and** ***V*** ^**2**^ **in electron-only devices with different film.**

The electron-extraction ability by the different film may be significantly dependent on the interface contact property and the energy-level alignment. To precisely assess the correlation among the factors, we have carefully examined the *μ*_e_ of electron-only devices with different film by using the SCLC method. In the work, the electron-only devices with an architecture of ITO/CBL/P3HT:ICBA/CsO_x_/Al were fabricated to measure the *μ*_e_. In such electron-only devices, the TiO_x_, CsO_x_, and TiO_x_/CsO_x_ is used as a CBL, whereas the CsO_x_ layer on the active layer surface is employed as a hole-blocking layer. Note that it is spin coated with Cs_2_CO_3_ solution and then thermally annealed for 160°C for 10 min in glove box filled with Ar. Generally, high performance of PSCs commonly accompanies with a high-electron mobility, which is mainly influenced by exciton dissociation, as well as charge-carrier recombination at the interfaces of donor/acceptor and the interfaces of CBL/acceptor. The single-carrier mobility can be obtained from the *J*_SCLC_*-V*^2^ curve (Figure 6) by the SCLC method using the Mott-Gurney square law [41]:

1 $$ {J}_{\mathrm{SCLC}}=\frac{9}{8}{\varepsilon}_r{\varepsilon}_0\mu \frac{V^2}{L^3} $$

where *ε*_r_ is the dielectric constant of the material; *ε*_0_ is the permittivity of free space; *L* is the distance between the cathode and anode, which is equivalent to the film thickness; and *V* is the applied voltage. Figure 6 shows the SCLC curves of the P3HT:ICBA-based electron-only devices with different film. It demonstrates the corresponding relationship of *J*_SCLC_ with *V*^2^, where *J*_SCLC_ is the dark current density. The *μ*_e_ of the devices with CsO_x_ film is only 3.85 × 10^−3^ cm^2^ V^−1^·s^−1^. Compared with the device with a CsO_x_ film, however, the devices with TiO_x_/CsO_x_ film show an enhanced remarkable *μ*_e_ of 5.52 × 10^−3^ cm^2^ V^−1^·s^−1^, suggesting an enhanced electron-extraction ability by the TiO_x_/CsO_x_ film and hence leading to the enhancement in *J*_SC_ and PCE of the inverted PSCs. It should be noted that the *μ*_e_ values of the inverted PSCs were higher than those of normal PSCs in previous work [42]. The data are summarized in Table 1. In the work, the increase of the *μ*_e_ should be also related to the reduction in work function of the TiO_x_ surface modified by CsO_x_. Li et al. reckoned that a thin layer of CsO_x_ is capable of lowering the work function of the underlying layer of ITO [27]. Moreover, Xu et al. reported inverted PSCs with a component film of Cs_2_CO_3_:4,7-Diphenyl-1,10-phenanthroline (BPhen) [43]. The work function of pristine BPhen on the ITO substrate was determined to be 3.1 eV by ultraviolet photoelectron spectroscopy, while the corresponding work function of the Cs_2_CO_3_:BPhen component layer was reduced to about 2.6 eV, thereby leading an increase in *V*_OC_ from 0.40 to 0.64 V and *J*_SC_ from 7.3 to 9.4 mA cm^−2^ of inverted PSCs with Cs_2_CO_3_:BPhen film as compared to inverted PSCs with BPhen film [43]. Combining all the above and our mentioned results, it is believed that the CsO_x_ (or Cs_2_CO_3_)-modified film can reduce the WF of the film and provide a better wetting property of the blend solvent on the TiO_x_/CsO_x_ film surface, as well as a favorable energy-level alignment, which facilitate electron injection from electron acceptor to cathode, and thus leading to a remarkable improvement in *V*_OC_ and *J*_SC_.

## Conclusions

In summary, high-efficiency inverted polymer solar cells are demonstrated with a solution-processed TiO_x_/CsO_x_ layer as a cathode buffer layer. By inserting a CsO_x_ film at the interface of the TiO_x_/active layer, the power conversion efficiency up to 5.65% and 3.76% has been achieved in inverted PSCs with P3HT:ICBA and inverted PSCs with P3HT:PCBM, respectively, under 100-mW cm^−2^ AM 1.5 G simulated solar illumination, suggesting that the TiO_x_/CsO_x_ is superior than the TiO_x_ and the CsO_x_. Moreover, this work not only provides a new option for the selection of the solution-processed cathode buffer layer in designing efficient and stable inverted PSCs, but also presents that the improvement of the interface contact property is also an essential factor for efficient polymer solar cells when preparing cathode buffer layers.
